# *BAP1* Status Determines the Sensitivity of Malignant Mesothelioma Cells to Gemcitabine Treatment

**DOI:** 10.3390/ijms20020429

**Published:** 2019-01-19

**Authors:** Alice Guazzelli, Parisa Meysami, Emyr Bakker, Constantinos Demonacos, Antonio Giordano, Marija Krstic-Demonacos, Luciano Mutti

**Affiliations:** 1 School of Environment and Life Sciences, University of Salford, Salford M5 4WT, UK; a.guazzelli@edu.salford.ac.uk (A.G.); p.meysami@salford.ac.uk (P.M.); m.krstic-demonacos@salford.ac.uk (M.K.-D.); 2 School of Medicine, University of Central Lancashire, Preston PR1 2HE, UK; ebakker@uclan.ac.uk; 3 Faculty of Biology, Medicine and Health, School of Health Sciences, University of Manchester, Manchester M13 9PL, UK; constantinos.demonacos@manchester.ac.uk; 4 Department of Medicine, Surgery and Neuroscience, University of Siena, 53100 Siena, Italy; antonio.giordano@unisi.it; 5 Sbarro Institute for Cancer Research and Molecular Medicine, Center for Biotechnology, College of Science and Technology, Temple University, Philadelphia, PA 19122, USA

**Keywords:** malignant mesothelioma, *BAP1*, gemcitabine, chemoresistance, DNA damage, cell cycle, apoptosis

## Abstract

Malignant mesothelioma (MMe) is a cancer with poor prognosis and resistance to standard treatments. Recent reports have highlighted the role of the *BRCA1 associated protein 1 gene* (*BAP1*) in the development of MMe. In this study, the chemosensitivity of human mesothelioma cell lines carrying *BAP1* wild-type (WT), mutant and silenced was analysed. The *BAP1* mutant cells were significantly less sensitive than *BAP1* WT cell lines to the clinically relevant drug gemcitabine. Silencing of *BAP1* significantly increased resistance of MMe cells to gemcitabine. Cell cycle analysis suggested that gemcitabine induced Sub-G1 phase accumulation of the *BAP1* WT cells and increased in the S-phase in both *BAP1* WT and mutant cells. Analysis of the role of BAP1 in apoptosis suggested that gemcitabine induced early apoptosis in both *BAP1* WT and *BAP1* mutant cells but with a much higher degree in the WT cells. Effects on the population of cells in late apoptosis, which can mark necrosis and necroptosis, could not be seen in the mutant cells, highlighting the possibility that BAP1 plays a role in several types of cell death. Significantly decreased DNA damage in the form of double-strand breaks was observed in gemcitabine-treated *BAP1* mutant cells, compared to *BAP1* WT cells under the same conditions. After *BAP1* silencing, a significant decrease in DNA damage in the form of double-strand breaks was observed compared to cells transfected with scramble siRNA. Taken together, the results presented in this manuscript shed light on the role of BAP1 in the response of MMe cells to gemcitabine treatment and in particular in the control of the DNA damage response, therefore providing a potential route for more efficient MMe therapy.

## 1. Introduction

Malignant mesothelioma (MMe) is a highly aggressive cancer, mostly related to asbestos exposure, genetics, other co-factors and gene x environment interaction with a poor prognosis and weak response to treatments [1,2,3,4,5,6]. Among the most important clinical characteristics of MMe is its resistance to standard chemotherapy. Several clinical trials assessed the use of chemotherapy as a single agent or in combination therapy including gemcitabine, a DNA damaging chemotherapy agent. Gemcitabine has been investigated as a first-line single treatment in chemotherapy-naive patients or as second- or third-line combination therapy in MMe patients. The results showed a low or moderate activity in patients with MMe [7,8,9,10,11]. Thus, it is imperative to achieve a better understanding of the biology and genetics of this tumour to design more specific, “personalised” treatments and/or overcome this resistance. Among the latest advances towards better understanding of this tumour, the discovery of *BAP1* gene mutations in MMe cells is one of the most intriguing due to potential translational implications [12,13,14,15]. 

BAP1 is a deubiquitinase enzyme, a member of the ubiquitin carboxy (C)-terminal hydrolase (UCH) family, involved in the regulation of cellular pathways such as the cell cycle, cellular differentiation, cell death, metabolism, and the DNA damage response [16,17,18]. BAP1 is involved in transcriptional regulation and has been found in complex with the host cell factor-1 (HCF-1) and the Yin Yang 1 (YY1) transcriptional regulators known to control chromatin modifications leading to both gene activation and repression [19]. BAP1/HCF-1 interaction is important for growth suppression in renal cancer; however, whether this is through BAP1-mediated deubiquitination and alteration of HCF-1 protein stability remains unclear [13,20].

Knockout of *BAP1* in HeLa cervical cancer and renal cancer cells exposed to ionising radiation resulted in increased cell death [13,21]. However, lack of BAP1 did not change the process of double-strand break repair [13,22], whilst the transcriptional profile of genes that control the DNA damage response was altered [16]. Although the exact role of BAP1 in cell cycle control and the DNA damage response and repair is not clear, some reports have suggested that BAP1 activity is controlled at various levels such as subcellular location and post-translational (PTM) modifications. In particular, the phosphatidylinositol 3-kinase-related kinases ATM/ATR/DNA-PK phosphorylate BAP1 at S592, which is one of the five serines in its carboxyl terminus that are modified in response to DNA damage [23,24,25,26]. Therefore, it is possible that upon DNA damage, BAP1 is phosphorylated and its function modified to mediate growth suppression.

Loss of *BAP1* due to mutations and deletions has been reported in various cancers including lung, renal, breast, uveal melanoma, and MMe [27]. In 2011 Bott et al. [28] reported somatic *BAP1* mutations in malignant pleural mesothelioma and Testa et al [14] also found MMe patients with germline *BAP1* mutations in the same year. Individuals that inherit one inactive *BAP1* allele (BAP1 tumour predisposition syndrome) have significantly higher predisposition to cancer [29,30,31]. *BAP1* mutations are associated with worse prognosis in uveal and cutaneous melanoma and renal cell carcinoma whereas they mark better outcomes for MMe patients [31]. Somatic *BAP1* point mutations were found in up to 60% of sporadic MMe [28,32,33,34].

The aim of this study is to investigate the potential link between BAP1 status and changes of sensitivity to a DNA damaging agent widely used as second line therapy in MMe [3,35]. The findings of this research are of high significance for clinical practice as they could be used to stratify MMe patients prior to treatment and avoid the use of a toxic drug as second line therapy that is unlikely to be effective in *BAP1* mutant patients.

Here, evidence has been provided that supports the view that BAP1 inactivation in MMe cells confers resistance to gemcitabine and provides further insight into the role of BAP1 in the cell cycle, cell death and DNA repair mechanisms in MMe cells.

## 2. Results

### 2.1. BAP1 WT MMe Cells Exhibit Higher Sensitivity to Gemcitabine Treatment Comprared to Mutated BAP1 MMe Cells

**Figure 1:**
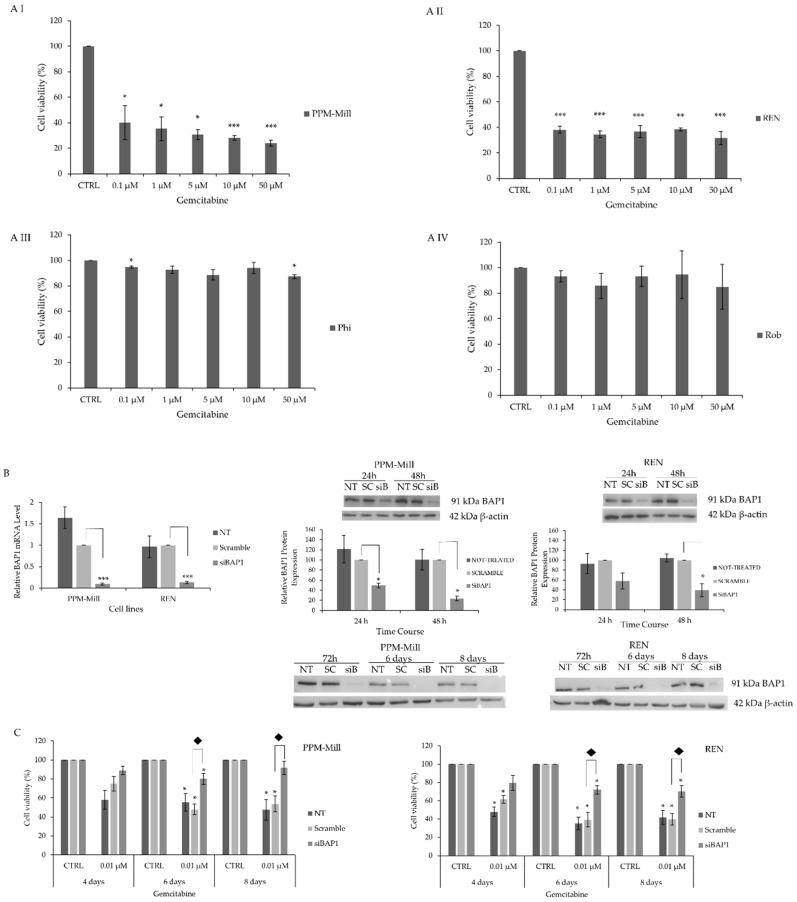
BRCA1 associated protein 1 (BAP1) modulates chemosensitivity of malignant mesothelioma (Mme). Sulphorhodamine B (SRB) proliferation assay in PPM-Mill (**A** I), REN (**A** II), Phi (**A** III) and Rob (**A** IV) cells treated with gemcitabine for 48 h at the indicated concentrations. qRT-PCR and Western blot analysis of PPM-Mill and REN cells treated with scramble and small interfering RNA (siRNA) targeting *BAP1* (**B**). SRB proliferation assay of PPM-Mill and REN cells either treated with 0.01 µM of gemcitabine or control (CTRL) treated with dimethyl sulfoxide (DMSO) that was used as vehicle in combination with the scramble and siRNA targeting *BAP1* for four, six, and eight days (**C**). Statistical analysis is described in Materials and Methods section. * *p* ≤ 0.05, ** *p* ≤ 0.01, *** *p* ≤ 0.001.

Given the importance of BAP1 in MMe, its potential involvement in chemosensitivity was investigated. Gemcitabine as a conventional treatment was used to assess its cytotoxic effect in *BAP1* WT and mutated cell lines. Cell viability of *BAP1* WT PPM-Mill and REN was significantly reduced by gemcitabine treatment (Figure 1A, I and II panels) compared to Phi and Rob which bear mutated *BAP1* (Figure 1A, III and IV panels). Cell viability of PPM-Mill and REN was reduced by approximately 60% at 0.1 µM of gemcitabine (statistically significant, *p* < 0.05 and *p* < 0.01 in PPM-Mill and REN, respectively) compared to control sample (CTRL), while cell viability of Phi and Rob was only slightly reduced by gemcitabine at all tested concentrations, thus showing a poor response. Silencing of BAP1 expression in *BAP1* WT PPM-Mill and REN cells—demonstrated using Western blot analysis and qRT-PCR (Figure 1B)—led to a significant reduction in sensitivity to gemcitabine (Figure 1C).

### 2.2. BAP1 Affects Cell Cycle Progression in MMe Cells Following Gemcitabine Treatment

**Figure 2:**
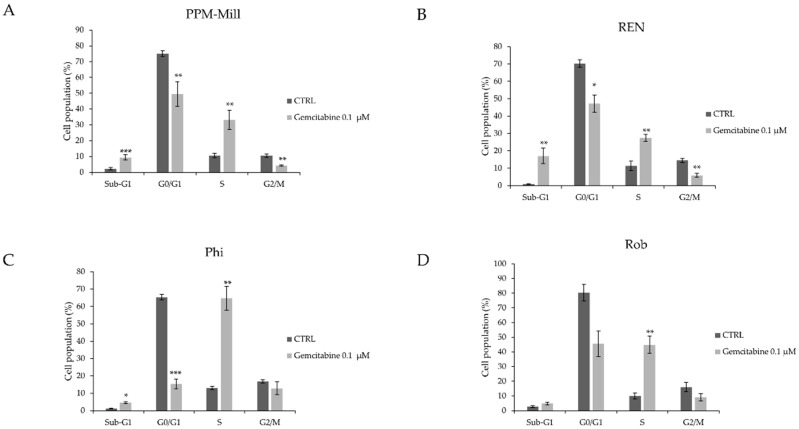
Cell cycle progression analysis in human mesothelioma cells with WT, mutated *BAP1* (**A**–**D**) treated with either 0.1 µM gemcitabine or with DMSO that was used as vehicle (CTRL) for 48 h, carried out using FACS). Statistical analysis is described in Materials and Methods section. * *p* ≤ 0.05, ** *p* ≤ 0.01, *** *p* ≤ 0.001.

To further investigate the role of BAP1 on the cell viability of mesothelioma cells treated with gemcitabine, cell cycle analysis was carried out. The PPM-Mill, REN, Phi, and Rob cell lines were treated with 0.1 µM gemcitabine for 48 h (Figure 2). Results demonstrated a significant increase of the percentage of cells in the Sub-G1 phase after gemcitabine treatment for PPM-Mill (Figure 2A) and REN (Figure 2B) cell lines (*BAP1* WT) to a greater level than in Phi (Figure 2C) and Rob (Figure 2D) cells (*BAP1* mutant) (Figure 2, compare Sub-G1 phase cell populations). The G1-phase declined in all cell lines irrespective of BAP1 status, but the extent varied depending on the cell type (Figure 2, compare bars G0/G1). Percentage of cells in the S-phase increased after gemcitabine treatment in all cell lines. The G2/M cell population decreased after gemcitabine treatment in all cell types (Figure 2, compare bars G2/M). Notably, these results indicate that gemcitabine induced more cell death in the *BAP1* WT cells compared to *BAP1* mutant cells.

### 2.3. BAP1 Status Affects Apoptotic Response to Gemcitabine Treatment

**Figure 3:**
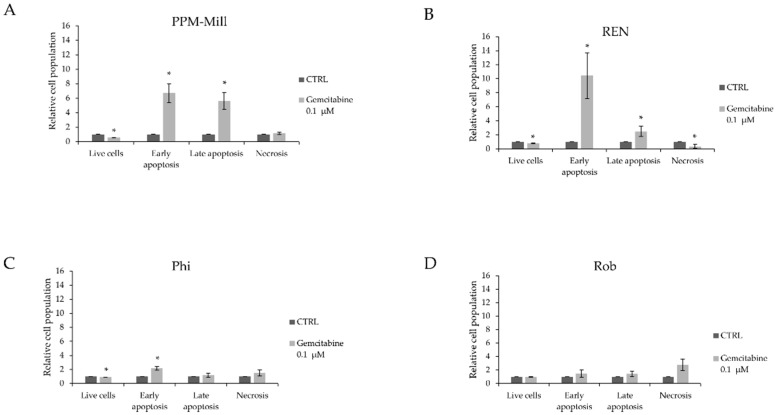
Annexin V assay in PPM-Mill, REN, Phi and Rob cells (**A**–**D**) treated with either 0.1 µM gemcitabine or DMSO that was used as vehicle (CTRL), for 48 h, conducted using FACS. Statistical analysis is described in Materials and Methods section. * *p* ≤ 0.05.

Cellular death occurs through multiple mechanisms including apoptosis, necrosis, and necroptosis. To determine the pathway by which gemcitabine induces cell death, the Annexin V assay, which measures apoptosis, was used. Gemcitabine treatment resulted in approximately 7-fold and 9-fold increase in early apoptosis in PPM-Mill and REN cells, respectively (Figure 3A,B), and approximately 3-fold increase in Phi cells (Figure 3C). Late apoptotic cell population significantly increased by approximately 6-fold in PPM-Mill cells and 2-fold in REN cells, whereas there was no significant increase in Phi and Rob cell lines (Figure 3A,B, compared to Figure 3C,D). Results shown in Figure 3 imply that functional BAP1 is important for the execution of both early and late apoptosis in response to gemcitabine; however, it seems to have a cell-specific effect in terms of the magnitude of its effect, as increased gemcitabine-mediated late apoptosis was evident in the *BAP1* WT cells only.

### 2.4. Gemcitabine Induces More DNA Damage in WT BAP1 than in BAP1-Mutated or -Silenced Cell Lines

**Figure 4:**
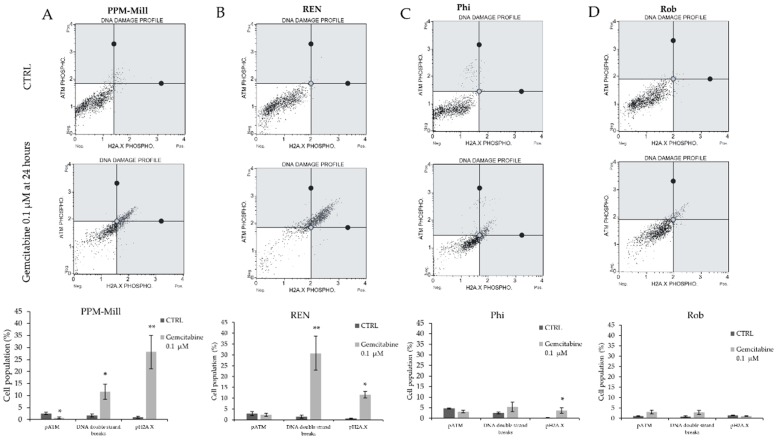

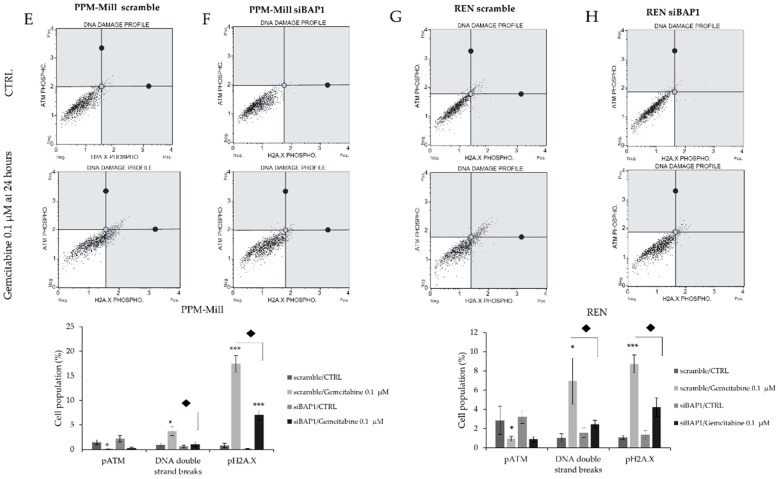
DNA damage assay in PPM-Mill, REN, Phi, and ROB un-transfected cells (**A**–**D**) or transfected with either scramble or siRNA targeting *BAP1* PPM-Mill and REN cells (**E**–**H**). All cells were either treated with 0.1 µM gemcitabine or DMSO that was used as vehicle (CTRL). The analysis was performed using Muse Analyser. Statistical analysis is described in Materials and Methods section. * *p* ≤ 0.05, ** *p* ≤ 0.01, *** *p* ≤ 0.001.

Activation of the DNA damage response can be measured by the phosphorylation of histone H2A.X. DNA damage was assessed in *BAP1* WT (Figure 4A,B) and *BAP1* mutated cell lines (Figure 4C,D) treated with gemcitabine (0.1 µM) for 24 h. Increased DNA double-strand breaks and γ-Η2Α.Χ phosphorylation were evident in gemcitabine-treated compared to non-treated PPM-Mill cells, whereas no ATM phosphorylation was detected in these cells under the same conditions. Increased DNA double-strand breaks and γ-Η2Α.Χ phosphorylation were recorded in gemcitabine-treated REN cells compared to untreated cells. *BAP1* WT cell lines transfected with siRNA targeting *BAP1* in combination with gemcitabine (0.1 µM) treatment for 24 h exhibited a significantly decreased percentage of DNA double-strand breaks and γ-Η2Α.Χ phosphorylation compared to scramble transfected (non-targeting siRNA) (Figure 4E–H) and *BAP1* WT cells.

## 3. Discussion

In this study, the role of BAP1 in the response to gemcitabine in MMe cells was investigated. Malignant mesothelioma cells with functional *BAP1* were more sensitive to gemcitabine treatment compared to cells bearing mutated and non-functional *BAP1*. The results obtained from the analysis of cell viability indicate that functional *BAP1* results in a better response to gemcitabine, that its status differentially affects the cell cycle progression in gemcitabine-treated versus non-treated cells and that BAP1 inactivation is linked to decreased DNA damage response after gemcitabine treatment.

Gemcitabine inhibits DNA synthesis through a mechanism known as “masked chain-termination” [36]. Resistance to gemcitabine treatment can be mediated by limited cellular uptake of this pyrimidine analogue due to NF-κB-dependent repression of the expression of the human concentrative nucleoside transporter 1 (hCNT1) which is the main transporter involved in gemcitabine cellular internalization [37]. BAP1 has been shown to indirectly suppress NF-κB binding to its target DNA sequences by inducing the expression of the transcription elongation factor A-like 7 (TCEAL7) [38]. Increased NF-κB transcriptional activity and consequent repression of hCNT1 expression in *BAP1* defective cells offers a potential explanation of the observed gemcitabine resistance in *BAP1* mutant cells.

Our results also demonstrated differential effects on cell cycle progression of MMe cells depending on their BAP1 status (Figure 2). Gemcitabine induces S-phase arrest [36,37,39] in MMe cell lines. Altered levels of BAP1 have been described to affect cell cycle progression. BAP1 was reported to regulate breast, lung and uterine cell proliferation and overexpression of BAP1 mostly inhibited cell cycle progression [40]. It interacts with and deubiquitinates the transcriptional regulator HCF-1. HCF1 deubiquitination affects the recruitment of histone methyltransferases to E2F1 target genes leading to alteration of the G1/S transition and transcription of genes required for S-phase [40]. In uveal melanoma cells, *BAP1* knockdown causes G1 arrest most likely through HCF1-mediated effects that involve histone deubiquitination and effects on E2F1-dependent transcription. BAP1 is also phosphorylated at serine 592 in response to DNA damage. Phosphorylation of BAP1 at serine 592 in response to DNA damage by ATM/ATR kinases retains BAP1 in the nucleus; however, it is predominantly dissociated from chromatin, suggesting that this phosphorylation removes BAP1 from specific promoters [26].

However, BAP1 has been reported to deubiquitinate KLF5 and to promote breast cancer cell proliferation and metastasis [41] and to play a pro-survival role in cutaneous melanoma [42]. In uveal melanoma cells, depletion of BAP1 resulted in a 20–40% reduction in cell cycle progression; however, it seems that this change was of a transient nature [43]. HeLa cells with inhibited BAP1 expression progressed through S-phase slower than control cells [44]. BAP1 also regulates mitotic spindle organization and prevents genomic instability by deubiquitinating γ-tubulin in breast cancer cells [45].

The results described in Figure 3 suggest that BAP1 is important for the execution of both early and late apoptosis in response to gemcitabine treatment. The role of BAP1 in regulating Ca^2^+ mediated apoptosis was discovered by Bononi et al. [46]. The role of BAP1 in apoptosis was also suggested by Ventii et al. [47] and it was hypothesised that BAP1 was required for both early and late apoptosis. However, further analysis including a Caspase-3 assay showed that BAP1 does not induce this marker of apoptosis, raising the possibility that it either has specific effect on the cell cycle by accelerating the progression through the G1/S checkpoint and/or triggering other types of cell death. It is intriguing therefore to hypothesise that BAP1 could cause cell death through apoptosis, necrosis or necroptosis which needs to be investigated further in order to evaluate potential therapeutic targets involved in this pathway such as RIPK1, which can be inhibited through Necrostatin-1 [48]. However, BAP1 was also reported to inhibit apoptosis induced as a result of glucose deprivation, highlighting the complexity of the function this protein plays in determining cell fate [49,50]. A novel mechanism by which BAP1 regulates apoptosis has been reported by Sime et al. [51] who demonstrated that the association between BAP1 and 14-3-3 protein releases the apoptotic inducer protein Bax from 14-3-3 and promotes cell death through the intrinsic apoptotic pathway.

It has been reported that BAP1 is recruited to the sites of DNA damage to promote DNA repair and that chicken lymphoma DT40 cells lacking BAP1 are more sensitive to ionizing radiation [16]. *BAP1*-deficient renal cell carcinoma cells were more sensitive to ionizing radiation than the *BAP1* WT cells, although this difference was marginal [21]. In cholangiocarcinoma, low BAP1 status conferred greater sensitivity to gemcitabine [52]. The results presented herein demonstrate that in *BAP1* WT cells gemcitabine induced an increase in DNA double strand breaks, whereas in cells with mutant *BAP1* gemcitabine did not have the same effect. These differences are potentially due to specific dual role that BAP1 has in mesothelioma compared to other types of cancer, where *BAP1* mutations increase predisposition to this cancer, but certain mutations can be associated with longer survival. These results are consistent with those of Bononi et al. [46], who reported that reduced levels of BAP1 in fibroblasts lead to lower ability to repair the DNA damage and increased survival of these cells after exposure to ionizing radiation.

Taken together, these results provide insight into the role of BAP1 with regard to drug resistance, cell cycle progression, apoptosis and DNA damage that may have potential translational implications. The most direct one is that a new way to stratify patients on BAP1 status is provided given the difference in sensitivity to chemotherapy. The augmented resistance of mutated *BAP1* cells seems to go against the clinical evidence that patients with MMe carrying *BAP1* mutations survive longer [53]. This apparent inconsistency could be due to the fact the *BAP1* WT promotes cancer stem cell generation (unpublished observations), which may help to explain the survival increase despite the decrease in chemosensitivity, in that the overall survival benefit that is observed is due to the lack of functional BAP1 driving cancer stem cell generation. The different sensitivity to DNA damage between *BAP1* mutant and WT also suggests BAP1 status could be the basis of selection of patients for treatment with poly ADP ribose polymerase (PARP) inhibitors, given that patients with *BAP1* mutated or *BAP1* WT (less sensitive and more sensitive to DNA damage respectively) are likely to respond differently to this type of inhibitors. Finally, it has been already proposed that defective DNA repair leads to chromosomal instability and higher mutational load [46,54], which potentially provides a rationale for patient stratification with regard to immunotherapy, according to BAP1 status. These findings raise questions about the controversial role of BAP1 in chemotherapy resistance and cancer cell survival. The mechanisms explaining the positive effects of *BAP1* mutations on survival are currently being studied in our laboratory. The preliminary results are indicative of the potential importance of metabolic reprogramming of cancer stem cells. The results of this study provide new insight into BAP1 status and changes of sensitivity to the DNA damaging agent gemcitabine, widely used in second-line therapy for MMe. The therapeutic options in this setting are very limited and demonstrate poor efficacy, which highlights the pivotal role of patient stratification. Further studies are needed to confirm the role of BAP1 status on chemosensitivity of MMe to other drugs used for this tumor such as pemetrexed and platinum-based treatments, as well as potential effects of gemcitabine on BAP1 signal transduction.

## 4. Materials and Methods

### 4.1. Cell Culture

Several human MMe cell lines were used, including MMe PPM-Mill (*BAP1* WT), MMe REN (*BAP1* WT), pleural MMe Phi (mutated *BAP1* with shorter splicing isoform) and Rob (*BAP1* null) [46], established by Pass et al. [55]. These cell lines were maintained in Dulbecco’s modified eagle medium (DMEM) with L-glutamine (Lonza, Nottingham, UK), supplemented with 10% Fetal Bovine Serum (FBS) (Life Technologies, Nottingham, UK), and 100 units/ml penicillin and 100 µg/mL of streptomycin (Lonza, UK) maintained at 37 °C in a 5% CO_2_ humidified atmosphere.

### 4.2. Treatment

Cells were treated with gemcitabine that was purchased from Sigma–Aldrich, Dorset, UK. Gemcitabine was stored at −20 °C at a stock concentration of 100 mM in DMSO, whilst the working concentration ranged between 50 µM and 0.1 µM.

### 4.3. Sulphorhodamine B (SRB) Assay

Cells were plated at density of 5 × 10^3^ cells/well in a 96-well plate 24 h before treating cells with the desired drugs. After treatment, cells were fixed with 10% trichloroacetic acid (TCA) for 1 h, and dried overnight at room temperature followed by staining with SRB for 15 min, washed twice with 1% acetic acid, and air dried for at least 1 h. The incorporated SRB staining was dissolved in 10 mM Tris pH 8.8 solution and then plates were analyzed using a calorimetric microplate reader (Thermo Electron Multiskan Ascent Microplate Reader) (Akribis Scientific, Knutsford, UK) at a wavelength of 540 nm and 690 nm.

### 4.4. BAP1 Silencing

SMARTpool: siGENOME Human BAP1 siRNA targeting BAP1 mRNA using a cocktail of a mixture of 4 siRNAs was purchased from Dharmacon, UK. SiGENOME Non-Targeting siRNA Pool #1 was used as non-targeting siRNA (scramble). SiRNA oligonucleotides were re-suspended in the provided buffer at a final stock concentration of 20 µM, siRNA transfection was performed using DharmaFECT 1 transfection reagent (Dharmacon GE, Cambridge, UK), according to the manufacturer’s instructions for reserve transfection for 24, 48, and 72 h, and six and eight days.

### 4.5. Quantitative Real Time-Polymerase Chain Reaction (qRT-PCR)

Total RNA was isolated by TRIZOL reagent (Sigma–Aldrich, Dorset, UK), according to the manufacturer’s instructions. Concentration and purity of RNA were determined using NanoDrop 2000 (Thermo Fisher Scientific, Loughborough, UK). Reverse transcription was performed with the iSCRIPT cDNA Synthesis Kit (Bio-Rad, Watford, UK) using 1 µg of total RNA. qRT-PCR was conducted using TaqMan^®^ Assay (Life Technologies, Nottingham, UK)). Relative expression levels of BAP1 mRNA were quantified using *18S ribosomal RNA* as a housekeeping gene and qRT-PCR was performed in Rotor-Gene Q (Qiagen, Manchester, UK), using pre-designed TaqMan probes.

### 4.6. Western Blot

Cells were collected in ice-cold RIPA buffer containing 1 mM DTT, 1 mM PMSF, 2 mM NaOV, 20 mM BGP and 5 mM NaPPi and 1 µg/mL protease and phosphatase inhibitors (Sigma, Dorset, UK). Protein concentrations were determined by the Bradford assay (Sigma, UK) and 30 µg of protein per well was loaded into sodium dodecyl sulfate (SDS) polyacrylamide gel. Proteins were transferred to the PDVF membrane. Membranes were blocked overnight via incubation at 4 degrees with 5% non-fat dry milk in phosphate-buffered saline (PBS). The membranes were treated with primary and secondary antibodies and blots developed using ECL substrate according to manufacturer’s instructions (Pierce, Fisher Scientific-UK Ltd., Loughborough, UK). The following antibodies were used for Western blotting: β-Actin (ab8227, Abcam, Cambridge, UK) and BAP-1 (sc-28383, Santa-Cruz Biotechnology, Middlesex, UK).

### 4.7. Cell Cycle Analysis

Cells were seeded in 6-well plates and treated with indicated drugs for 48 h. Cells were detached from the plate and collected using centrifugation at 300× *g* for 5 min. Pellets were washed with PBS before adding 1 mL of 70% EtOH drop-wise. After washing with PBS, 50 µL of RNase (100 µg/mL) was incubated at 37 °C in the dark for 15 min, after which 300 µL of 50 µg/mL propidium iodide (PI) solution was added. The samples were then processed using a BD FACSVerse™ flow cytometer and analyzed using BD FACSuite™ software (Berkshire, UK).

### 4.8. Annexin V Staining

For the analysis of apoptosis, cells were seeded at a cell density of 2.5 × 10^4^ cell/mL. After 48 h of treatment, cells were collected and resuspended in the binding buffer and stained using a fluorescent labelled Annexin V:FITC for 10 min in the dark and in combination with propidium iodide solution according to manufacturer’s instructions. The samples were processed using FACSVerse™ flow cytometer (Berkshire, UK) and analyzed using BD FACSuite™ software.

### 4.9. Multi-Color DNA Damage Assay

To assess DNA damage, 10 × 10^4^ cells/well were seeded in 6-well plates and treated with indicated drugs for 24 h. Cells were fixed and stained with anti-phosphor Histone H2A.X (Ser139) and anti-phosphor ATM (Ser1981) antibodies according to manufacturer’s instructions (Muse Multi-Color DNA Damage Kit (Merck Millipore, Watford, UK)). The samples were analyzed using MuseTM Cell Analyser (Watford, UK).

### 4.10. Statistical Analysis

All data are representative of at least two independent experiments. Error bars represent standard error of means. *p*-value ≤ 0.05, 0.01, and 0.001 is indicated by *, **, and ***, respectively. A paired, two-tail student’s *t*-test was conducted comparing samples to the control for statistical significance analysis. Diamond indicates statistical significance when siRNA-treated samples were compared to scramble-treated cells.

## Abbreviations

ATM	Ataxia-Telangiectasia-Mutated
ATR	Ataxia Telangiectasia and Rad3-related
BAP1	BRCA associated protein 1
DNA-PK	DNA-dependent Protein Kinase
E2F1	E2F transcription factor 1
GEM	Gemcitabine
HCF-1	Host Cell Factor-1
hCNT1	human Concentrative Nucleoside Transporter
KLF5	Krueppel-Like Factor 5
MMe	Malignant Mesothelioma
NF-κB	Nuclear Factor kappa-light-chain-enhancer of activated B cells
OGT	O-linked N-acetylglucosamine transferase
PRC1	Polycomb repressive complex 1
PR-DUB	Polycomb repressive deubiquitinase
TCEAL7	Transcription Elongation factor A-like 7
UCH	Ubiquitin carboxy (C)-terminal Hydrolase
WT	Wild Type
YY1	Yin Yang 1
